# STI prevalence and the integration of point-of-care nucleic acid amplification testing into STI diagnostic algorithms at a Médecins Sans Frontières key population clinic in San Pedro Sula, Honduras

**DOI:** 10.3389/frph.2026.1685453

**Published:** 2026-02-23

**Authors:** Derek C. Johnson, Kimberly Rodriguez, Diana Gómez-López, Darío Rodríguez, Diana Dávila, Lindsay Salem-Bango, Joaquim Guinart Verdaguer, Carina Perotti, Reinaldo Ortuño Gutiérrez, Nelly Staderini, Iza Ciglenecki

**Affiliations:** 1 Central America and Mexico Integrated Office, Médecins Sans Frontières, Mexico City, Mexico; 2 Project HN142, Médecins Sans Frontières, San Pedro Sula, Honduras; 3 Operational Center Geneva Headquarters, Médecins Sans Frontières, Geneva, Switzerland

**Keywords:** GeneXpert, Honduras, key population, LGBTQIA+, nucleic acid amplification testing (NAAT), point-of-care diagnostics, sex work, sexually transmitted infections (STI)

## Abstract

**Background:**

Data is limited on the prevalence of sexually transmitted infections (STIs) among key populations in Honduras. Additionally, clinics largely rely on syndromic management of STIs, which has poor diagnostic performance. This study assesses STI prevalence and the feasibility and diagnostic utility of rapid nucleic acid amplification testing (NAAT) in comparison to syndromic identification among the LGBTQIA+ and sex worker community of San Pedro Sula attending a Médecins sans Frontières (MSF) clinic.

**Methods:**

Patients attending MSF's San Pedro Sula clinic from February to June 2024, were invited to participate in the study. Clinicians assessed all participants for STI symptoms and, regardless of symptoms, collected whole blood, urine, and vaginal samples. Rapid testing [Human Immunodeficiency Virus (HIV), Hepatitis B (HBV), Hepatitis C (HCV), and syphilis] and NAAT via GeneXpert [chlamydia, gonorrhea, trichomoniasis, and human papilloma virus (HPV)] were performed. Treatment was initially prescribed per WHO Syndromic Management Guidelines and revised following NAAT results. Descriptive statistics and diagnostic metrics were calculated. Focus groups with clinic staff assessed feasibility.

**Results:**

Of the 157 patients enrolled, 31.8% (*n* = 50) tested positive for at least one STI: HPV 19.4% (7/36), syphilis 12.1% (19/157), chlamydia 10.2% (16/157), gonorrhea 8.3% (13/157), trichomoniasis 3.8% (6/157), HIV 3.8% (6/157), HBV 0% (0/157), and HCV 0% (0/157). Of those, 38.0% (*n* = 19) tested positive for multiple STIs. Only 29.3% (*n* = 46) of all participants and 56.6% (*n* = 22) of positive tests for chlamydia, gonorrhea, syphilis, or trichomoniasis were symptomatic. Staff felt GeneXpert benefited patient care but were concerned about sustainability.

**Conclusion:**

This study underscores the high STI prevalence among key populations in San Pedro Sula, Honduras. Results show that point-of-care NAAT implementation is beneficial, appreciated, and feasible in this context and can successfully be integrated into basic clinic diagnostics. The added testing capacity improved diagnostic and management capacity of the clinic, especially regarding asymptomatic STIs, and thus improved quality of care for key populations. A translated version of this manuscript in Spanish can be found in Supplementary Appendix S1.

## Introduction

According to the World Health Organization (WHO), more than one million curable sexually transmitted infections (STIs)—including chlamydia, gonorrhea, trichomoniasis, and syphilis—are contracted by individuals every day (1). STI rates are typically higher among key populations, who often face discrimination and lower access to care (2). In Honduras, sex workers and persons who are lesbian, gay, bisexual, transgender, queer, intersex, asexual, or with other sexual orientations or identities (LGBTQIA+) face considerable stigmatization and discrimination that impacts their access to sexual and reproductive health (SRH) services (3, 4). To reduce STI transmission and antimicrobial resistance, there are increasing efforts by the local government to standardize STI services particularly for high risk populations (5). Within this landscape, Médecins sans Frontières (MSF) opened a clinic specifically for LGBTQIA+ individuals and sex workers in San Pedro Sula, Honduras, in 2021. The clinic provides mental, sexual, and reproductive health services, in addition to linkages to social services. A health promotion team also conducts health education and community engagement.

Data is limited on current STI prevalence among key populations in Honduras. At the national level, STI data is not disaggregated by sexual orientation, gender identity, or sex worker status, limiting publicly available data. A 2010 study on STIs among men who have sex with men (MSM) in Honduras found that 37.6% reported an STI in the last year (6), and a 2006–2008 study on female sex workers found syphilis and chlamydia prevalence of 2.3% and 6.1%, respectively (7). However, this published literature is over 15 years old. This gap in updated STI prevalence hinders tailored programming and service delivery.

STI service delivery in Honduras is also hindered by limited molecular diagnostic capacity. Clinics in Honduras largely rely on syndromic management of STIs, or the diagnosis and treatment of potential STIs via symptoms only (5). While clinics can send samples to regional laboratories for molecular testing, logistical challenges like limited supplies and transportation delays mean that initial diagnosis and treatment are based on symptoms only, following WHO's symptomatic management guidelines (8). WHO recommends molecular testing like the nucleic-acid amplification test (NAAT)—widely considered to be the “gold-standard” for STI screening (9)—for proper STI diagnosis and treatment, but they provide syndromic management guidelines to support diagnosis and treatment when molecular testing is unavailable (8). While useful in low-resource settings, syndromic management has consistently been shown to have low sensitivity and specificity, which can result in misdiagnosis and mistreatment (9, 10). While exact proportions vary by STI and context (11), WHO recognizes that the majority of STI cases globally are asymptomatic (12). For example, studies have reported that 58.6–95.8% (median 75.0%) and 13.3–99.3% (median 65.0%) of *N. gonorrhoeae* cases and *C. trachomatis*, respectively, were asymptomatic (11). Cases that are symptomatic often have non-specific symptoms (12). Misdiagnosis of STIs can cause complications from untreated infections such as (but not limited to): low birth weight, pre-term birth, increased Human Immunodeficiency Virus (HIV) infectiousness and HIV susceptibility, cervical cancer, pelvic inflammatory disease, infertility, conjunctivitis neonatorum, and reactive arthritis (13–15)^,^. Moreover, misdiagnosis can lead to unnecessary or incorrect antibiotic use, which can contribute to antibiotic resistance of STIs (16, 17).

Advancements in molecular testing aid the accurate diagnosis and treatment of STIs. The GeneXpert assay (Cepheid, Sunnyvale, CA), a point-of-care diagnostic test based on real-time polymerase chain reaction (PCR) NAAT has been identified as a way to improve STI diagnostics and treatment. Previous feasibility studies show the GeneXpert assays being used in a variety of settings (18–20) with over 95% concordance when compared to conventional PCR methods (21).

To better understand the STI prevalence among key populations in Honduras and the feasibility of integrating GeneXpert to improve diagnostic capacity, a cross-sectional study was implemented within MSF's clinic in San Pedro Sula. A Spanish-translated version of this manuscript can be found in Supplementary Appendix S1.

## Methodology

### Study setting

This cross-sectional study occurred in an MSF-operated clinic in San Pedro Sula, Honduras, from February 19 to June 3, 2024. The clinic specializes in the provision of non-judgmental health care for LGBTQIA+ individuals and sex workers and staffs a nurse, physician, psychologist, social worker, and health promotion team. It primarily provides mental, sexual, and reproductive health services, although general health needs are also addressed. First-time patients who do not meet the target population are provided services and then linked to other facilities. Prior to the study, only rapid diagnostic tests for HIV and syphilis were provided on-site. Samples for molecular testing were sent off-site to the government-run regional laboratory.

### Participants

Individuals attending MSF's clinic in San Pedro Sula during the study period were first screened for eligibility using inclusion and exclusion criteria. If the individual was at least 18 years old, had engaged in sexual activity in the last 3 months, and had a working telephone number, they were approached by MSF-trained health staff who explained the study and helped facilitate the informed consent process. Only those who provided informed consent were enrolled in the study. To calculate sample size, estimated prevalences of chlamydia, gonorrhea, HPV, HIV, HBV, HCV, syphilis, and trichomoniasis were extracted from aforementioned scientific literature (6, 7). Sample size was calculated to a 4% precision with 95% confidence. Resulting sample sizes varied by STI and ranged from 57 to 316 participants: gonorrhea (*n* = 57); HCV (*n* = 61); HIV (*n* = 64); syphilis (*n* = 67); HBV (*n* = 88); trichomoniasis (*n* = 130); chlamydia (*n* = 145); and HPV (*n* = 316).

For qualitative procedures, patients enrolled in the study were invited to participate in an interview and a second consent was collected. Clinic staff were invited to participate in a focus group discussion (FGD), and informed consent was collected from those who agreed to participate. A flowchart of the study process can be found in Supplementary Appendix S2.

### Procedure

Each participant, regardless of reported symptoms, was evaluated by the clinic nurse and/or doctor for STIs using WHO's syndromic management approach. Assessed signs and symptoms included: dysuria, dyspareunia, vaginal discharge, genital ulcer, genital pain, or anal ulcer.

Regardless of the syndromic evaluation results, each participant underwent rapid tests for HIV, Hepatitis B (HBV), Hepatitis C (HCV), and syphilis. NAAT testing was conducted via GeneXpert for high-risk HPV genotypes, chlamydia, gonorrhea, and trichomoniasis. For NAAT testing, undiluted urine samples were collected for chlamydia, gonorrhea, and trichomoniasis; cervical-vaginal swabs for human papillomavirus (HPV) testing (for female participants) were either self-sampled or collected by a health provider, by patient preference. Urine samples that were not processed within the first 4 h after sampling were preserved using the CT/NG URINE-50 Urine Transport Medium Collection Kit. Samples for NAAT testing were processed on the GeneXpert system Cepheid 10-color Dx IV using GeneXpert software version 4.3. The system automates and integrates sample purification, nucleic acid amplification, and detection of the target sequences in samples using real-time PCR assays. Cepheid, the manufacturer of the GeneXpert system, has developed cartridges for the detection of 13 types of high-risk HPV, chlamydia, gonorrhea, trichomoniasis, and other pathogens from swabs or urine within 90 min (22). The GeneXpert system was located at the clinic, requiring a stable source of electricity for operation. A trained laboratory technician oversaw sample loading, system maintenance and result delivery to clinical staff.

5 mL of whole blood was collected for rapid testing of HIV, HBV, HCV, and Syphilis. Patients who received a positive result from a rapid test received a second rapid test during a follow-up appointment.

Initial STI treatment was provided following a syndromic approach, with medications prescribed according to MSF guidelines (23). Treatment was then updated as needed following NAAT testing. Results were typically available 1–2 h after the initial consultation; if the sample was collected at the end of the workday or week, results were available the next business day. Participants were offered the option to wait in the clinic for their results or to return later that day or the following day for results. If a participant did not return to the clinic, they were contacted via telephone and reminded to return for their results. Patients who received treatment for bacterial STIs were scheduled to come for a follow-up visit after 3 months for re-testing, and 6 months if they tested positive for syphilis. Additional non-treponemal tests were requested in an external laboratory for patients with both a current positive syphilis test and a history of previous syphilis infection. Patients who contacted the clinic for persistent symptoms one week after antibiotic treatment were asked to return for further assessment.

### Qualitative procedures

To assess feasibility, one FGD was conducted with implementing clinic staff (*n* = 8) one month before the end of the study. Questions focused on their experiences integrating GeneXpert into the clinic's STI testing schema. To include patient perspectives, 26 individual interviews were conducted among patient participants. Interviews were conducted in-person or via telephone per participant preference and availability. Questions centered around their perspectives on care provided by MSF, test availability, and sexual health needs. To reduce response bias, the interviews were conducted by research team members who were not in direct contact with patients, and the FGD was conducted by a researcher who was not part of the clinical staff. Both the FGD and interviews were conducted in Spanish; the quotes presented herein were translated to English. For both the FGD and interviews, thematic analyses were conducted to identify key themes among responses.

### Statistical analysis

STIs were classified into two categories for analysis based on WHO syndromic management guidelines (8): (1) “Any STI” which includes all STIs screened for in the study, and (2) “Antibiotic-treated STIs”, which included chlamydia, gonorrhea, trichomoniasis, and syphilis. Descriptive statistics and summary tables were used to describe the participant population and STI results. Indicators for determining STI prevalence were based on the number of positive and negative NAAT tests for each STI. Where appropriate, statistical significance was evaluated using univariate tests. For univariate testing, Pearson's Chi-squared and Fisher's exact tests were used to assess statistical significance between categorical variables. For univariate testing assessing statistical significance between categorical and continuous variables, the Wilcoxon Rank-Sum test was used. The sensitivity/specificity and Positive Predictive Value (PPV)/Negative Predictive Value (NPV) of syndromic testing were determined for testing positive for one or more of the following STIs: (1) chlamydia, (2) gonorrhea, (3) syphilis, (4) trichomoniasis, where testing positive for an STI using NAAT testing was regarded as a gold standard “true value” for STI positivity. Differences between the number of STI cases correctly identified through syndromic methods compared to NAAT testing was analyzed according to sex assigned at birth.

## Results

### Descriptive characteristics

From February 19 to June 3, 2024, 157 individuals were enrolled in the study. The median age was 29 years [Interquartile Range (IQR) 23–33 years] and 65.0% (*n* = 102) were biological males (Table 1). The most common gender identity was cisgender male (59.9%, *n* = 94) followed by cisgender female (36.3%, *n* = 57). Transgender females and non-binary individuals represented 3.1% (*n* = 5) of the population. There was a significant difference in sexual orientation between males assigned to birth and females assigned to birth, where the majority of males identified as homosexual (72.5%, *n* = 74) and the majority of females identified as heterosexual (63.6%, *n* = 35) (*p*-value < 0.001). Most study participants were single (74.5%, *n* = 118) or living with their partner but not married (20.4%, *n* = 32). There was a significant overall difference in marital status and biological sex, with more men than women reporting being single (82.0%, *n* = 84 vs. 60.0%, *n* = 33) and more women than men were living with their partner but not married (30.9%, *n* = 17 vs. 15.0%, *n* = 15). 26.8% of participants (*n* = 42) reported being previously diagnosed with an STI. Biological males were more than twice as likely to report a previous STI than biologic females (33.3%, *n* = 34 vs. 14.5%, *n* = 8; *p* < 0.05) (Table 1).

**Table 1:** Descriptive characteristics of participants stratified by biological Sex and the diagnosis of STIs.

Variable	Overall (*n* = 157)	Biological sex			Diagnosis of STI			
		Male (*n* = 102)	Female (*n* = 55)	*p*-value^d^	None (*n* = 107)	Any STI (*n* = 50)	Antibiotic-treated STI^e^ (*n* = 41)	*p*-value^d^
Demographics^a^								
Age^a^	29 (8)	29 (8)	29 (8)	0.7	28 (24, 33)	27 (21, 33)	26 (21, 33)	0.3
Biological sex^c^^,^^g^								
Male	102 (65.0%)	–	–		73 (68.2%)	29 (58.0%)	26 (63.1%)	0.3
Female	54 (35.0%)	–	–		34 (31.8%)	20 (40.0%)	15 (36.6%)	
Gender Identity^c^^,^^f^				<0.001***				0.6
Cisgender Male	94 (59.9%)	94 (92.1%)	0 (0.0%)		67 (62.6%)	27 (54.0%)	23 (56.1%)	
Cisgender Female	57 (36.3%)	2 (2.0%)	55 (100.0%)		37 (34.6%)	20 (40.0%)	15 (36.6%)	
Transgender Female	4 (2.5%)	4 (3.9%)	0 (0.0%)		2 (1.9%)	2 (4.0%)	2 (4.9%)	
Queer/Non-binary	1 (0.6%)	1 (1.0%)	0 (0.0%)		1 (0.9%)	0 (0.0%)	0 (0.0%)	
Sexual Orientation^c^^,^^f^				<0.001***				0.11
Heterosexual	37 (23.6%)	2 (2.0%)	35 (63.6%)		20 (18.7%)	17 (34.0%)	7 (17.1%)	
Gay/Homosexual	74 (47.1%)	74 (72.5%)	0 (0.0%)		55 (51.4%)	19 (38.0%)	16 (39.0%)	
Lesbian	9 (5.7%)	1 (1.0%)	8 (14.5%)		7 (6.5%)	2 (4.0%)	14 (34.1%)	
Bisexual	30 (19.7%)	19 (18.6%)	12 (21.8%)		22 (20.6%)	8 (16.0%)	1 (2.4%)	
Other	6 (3.8%)	6 (5.9%)	0 (0.0%)		3 (2.8%)	3 (6.0%)	3 (7.3%)	
Marital Status^c^				0.009**				0.3
Married	6 (3.8%)	2 (2.0%)	4 (7.3%)		4 (3.7%)	2 (4.0%)	2 (4.9%)	
Divorced	1 (0.6%)	1 (1.0%)	0 (0.0%)		1 (0.9%)	0 (0.0%)	0 (0.0%)	
Single	118 (74.5%)	84 (82.4%)	33 (60.0%)		84 (78.5%)	34 (68.0%)	29 (70.7%)	
Living with partner (not married)	32 (20.4%)	15 (14.7%)	17 (30.9%)		18 (16.8%)	14 (28.0%)	10 (24.4%)	
Any Children (Yes)^c^	43 (27.3%)	8 (7.8%)	35 (63.6%)	<0.001***	30 (28.0%)	13 (26.0%)	11 (26.8%)	0.56
STI History^c^								
Previous STI testing	132 (84.1%)	88 (86.3%)	44 (80.0%)	0.4	91 (85.0.%)	41 (82.0%)	34 (82.9%)	0.09
Previous STI diagnosis	42 (26.8%)	34 (33.3%)	8 (14.5%)	0.013*	22 (20.6%)	20 (40.0%)	17 (41.1%)	0.005**
Risk Factors								
Partner with STI in last 3 months (Yes)^c^	21 (13.4%)	17 (16.7%)	4 (7.3%)	0.095	15 (14.0%)	6 (12.0%)	5 (12.2%)	0.7
No. of sexual partners in last month^b^	1 (1, 4)	1 (1, 3)	2 (1, 9)	0.017*	1 (1, 3)	1 (1, 4)	1 (1, 5)	0.4
Current drug use^c^				0.3				
Any	33 (21.0%)	24 (23.5%)	9 (16.4%)		23 (21.5%	10 (20.0%)	7 (17.1%)	
Injectable	3 (1.9%)	2 (2.0%)	1 (1.8%)		2 (1.9%)	1 (2.0%)	0 (0.0%)	
Talk with partners about STI prevention^c^	101 (64.3%)	69 (67.6%)	32 (58.2%)	0.2	75 (70.1%)	27 (54.0%)	22 (53.7%)	0.046*
Frequency of condom use^c^				0.5				0.2
Always	43 (26.8%)	33 (32.4%)	10 (18.2%)		33 (30.8%)	10 (20.0%)	9 (22.0%)	
Often	13 (8.3%)	8 (7.8%)	5 (9.1%)		10 (9.3%)	3 (6.0%)	3 (7.3%)	
Sometimes	40 (25.5%)	26 (25.5%)	14 (25.5%)		28 (26.2%)	12 (24.0%)	9 (22.0%)	
Hardly Ever	2 (1.3%)	2 (2.0%)	0 (0.0%)		0 (0.0%)	2 (4.0%)	2 (4.9%)	
Never	4 (2.5%)	2 (2.0%)	2 (3.6%)		2 (1.9%)	2 (4.0%)	2 (4.9%)	

^a^ Demographic information was missing from 1 individual.

^b^ Median (IQR).

^c^ n (%).

^d^ Wilcoxon rank sum test; Fisher's exact test; Pearson's Chi-squared test.

^e^ Chlamydia, gonorrhea, trichomoniasis, and syphilis.

^f^ Gender identity and sexual orientation were self-identified.

^g^ For the statistical comparison of “Biological Sex” and “Any STI” and “Antibiotic-Treated STI”, only the STIs chlamydia, gonorrhea, and syphilis were included in the analysis.

* *p* < 0.05.

** *p* < 0.01.

*** *p* < 0.001.

### STI prevalence

31.8% of participants (*n* = 50) tested positive for at least one STI. STI percentages based on the total population as a denominator were as follows: HPV 19.4% (*n* = 7), syphilis 12.1% (*n* = 19), chlamydia 10.2% (*n* = 16), gonorrhea 8.3% (*n* = 13), trichomoniasis 3.8% (*n* = 6), HIV 3.8% (*n* = 6), HBV 0% (*n* = 0), and HCV 0% (*n* = 0) (Table 2). Of those diagnosed with an STI, 38% (*n* = 19) tested positive for more than one STI. Trichomoniasis was most likely to be accompanied by a coinfection (100.0% *n* = 6), followed by HIV (66.6%, *n* = 4), chlamydia (56.3%, *n* = 9), gonorrhea (53.8%, *n* = 7), syphilis (52.6%, *n* = 10), and HPV (28.6%, *n* = 2) (Table 3).

**Table 2:** STI diagnoses.

Variable	Any STI	No STI	Chlamydia	Gonorrhea	Syphilis	Trichomoniasis	HIV	HPV^&^	HBV	HCV
Positive^a^^,^^c^	50 (31.8%)	107 (68.2%)	16 (10.2%)	13 (8.3%)	19 (12.1%)	6 (3.8%)	6 (3.8%)	7 (19.4%)	0 (0.0%)	0 (0.0%)
Biological sex^c^^,^^d^										
Male	29 (58.0%)	73 (68.2%)	5 (31.3%)**	10 (76.9%)	17 (89.5%)*	–	5 (83.3%)	–	0 (0.0%)	0 (0.0%)
Female	20 (40.0%)	34 (31.8%)	11 (68.8%)	2 (15.4%)	2 (10.5%)	6 (100%)	1 (16.7%)	7 (19.4%)	0 (0.0%)	0 (0.0%)
Symptoms^b^^,^^c^										
Any	23 (46.0%)	23 (21.5%)	7 (43.8%)	12 (92.3%)	10 (52.6%)	1 (16.7%)	3 (50.0%)	0 (0.0%)	0 (0.0%)	0 (0.0%)
Dysuria	11 (22.0%)	7 (6.5%)	1 (6.3%)	9 (69.2%)	3 (15.8%)	1 (16.7%)	3 (50%)	0 (0.0%)	0 (0.0%)	0 (0.0%)
Dyspareunia	8 (16.0%)	6 (5.6%)	2 (12.6%)	4 (30.8%)	3 (15.8%)	1 (16.7%)	1 (16.7%)	0 (0.0%)	0 (0.0%)	0 (0.0%)
Vaginal discharge^e^	6 (30.0%)	5 (4.7%)	2 (12.6%)	4 (30.8%)	1 (5.3%)	0 (0.0%)	0 (0.0%)	0 (0.0%)	0 (0.0%)	0 (0.0%)
Genital ulcer	1 (2.0%)	0 (0.0%)	0 (0.0%)	0 (0.0%)	1 (5.3%)	0 (0.0%)	1 (16.7%)	0 (0.0%)	0 (0.0%)	0 (0.0%)
Anal ulcer	0 (0.0%)	1 (0.9%)	0 (0.0%)	0 (0.0%)	0 (0.0%)	0 (0.0%)	0 (0.0%)	0 (0.0%)	0 (0.0%)	0 (0.0%)
Genital vesicle/blister	0 (0.0%)	1 (0.9%)	0 (0.0%)	0 (0.0%)	0 (0.0%)	0 (0.0%)	0 (0.0%)	0 (0.0%)	0 (0.0%)	0 (0.0%)

^a^ Percentage tested positive was calculated using the number of total participants (*n* = 157) as the denominator.

^b^ Percentage with symptoms was calculated using the number of positive tests for the indicated STI as the denominator.

^c^ n (%).

^d^ Where appropriate, Wilcoxon rank sum test; Fisher's exact test; Pearson's Chi-squared test conducted on differences by sex.

^e^ Only biological women were taken into account.

* *p* < 0.05.

** *p* < 0.01.

*** *p* < 0.001.

^&^ Only 36 women received HPV screening.

**Table 3:** Number of STI co-infections.

Co-infection	Chlamydia (*N* = 9)	Gonorrhea (*N* = 7)	Syphilis (*N* = 10)	Trichomoniasis (*N* = 6)	HPV (*N* = 2)	HIV (*N* = 4)
**Chlamydia (*N* = 9)**	–	–	–	–	–	–
**Gonorrhea (*N* = 7)**	2	–	–	–	–	–
**Syphilis (*N* = 10)**	3	4	–	–	–	–
**Trichomoniasis (*N* = 6)**	3	0	1	–	–	–
**HPV (*N* = 2)**	1	0	0	1	–	–
**HIV (*N* = 4)**	0	1	2	1	0	–

Individuals with a previous STI diagnosis were more than twice as likely to test positive during their clinic visit than individuals who had never tested positive for an STI (40.0% vs. 20.6%) (Table 1). While there was no significant difference in overall STI incidence by sex, there was a significant difference for specific STIs. More men (16.6%) than women (3.2%) tested positive for syphilis (*p* < 0.05). Conversely, more women (17.5%) tested positive for chlamydia than men (4.9%) (*p* < 0.01).

### STI symptoms

29.5% (*n* = 46) of all participants reported STI symptoms, with dysuria being the most common (39.0% of people who reported symptoms, *n* = 18), followed by dyspareunia (30.0%, *n* = 14), and vaginal discharge (20.0%, *n* = 11 of women who reported symptoms) (Table 2). Of the 46 participants with STI symptoms, 50.0% (*n* = 23) subsequently tested positive for an STI. However, not all participants with an STI reported symptoms (54.0%, *n* = 27). Of positive NAAT tests for chlamydia, gonorrhea, syphilis, or trichomoniasis, only 56.6% had reported symptoms: gonorrhea 92.3% (12/13), syphilis 52.6% (10/19), chlamydia 43.8% (7/16), HIV 50% (3/6) and trichomoniasis 16.7% (1/6).

### Syndromic management vs. NAAT testing

The overall sensitivity and specificity of syndromic identification of STIs compared to NAAT screening was 53.7% (95% CI: 37.4–69.3%) and 78.5% (95% CI: 69.9–85.5%) respectively, with a PPV of 46.8% (95% CI: 35.9–58.0) and NPV of 82.7% (95% CI: 77.3–87.1%), and an accuracy of 71.9% (95% CI: 64.3%–78.8%) (Table 4). The sensitivity and specificity of specific symptoms for STIs, where discharge symptoms were used to assess gonorrhea, chlamydia, or trichmoniasis and ulcers were used to assess syphilis was 53.4% (95% CI: 31.8–60.7%) and 79.3% (95% CI: 70.8–86.3%), with a PPV of 47.8% (95% CI: 36.7–59.2%) and a NPV of 82.1% (95% CI: 76.8–86.5%) (Table 4). In both cases, men had better point estimates for sensitivity, specificity, PPV, and NPV than women regarding syndromic identification of STIs.

**Table 4:** Testing metrics.

Statistic	Overall (95% CI)	Men (95% CI)	Women (95% CI)
Syndromic management			
Sensitivity	53.7% (37.4%–69.3%)	48.4% (30.2%–66.9%)	46.7% (21.3%–73.4%)
Specificity	78.5% (69.9%–85.5%)	84.5% (73.9%–92.0%)	77.5% (61.6%–89.2%)
PPV	46.8% (35.9%–58.0%)	57.7% (41.5%–72.4%)	43.8% (26.1%–63.1%)
NPV	82.7% (77.3%–87.1%)	78.9% (72.5%–84.3%)	79.5% (70.1%–86.5%)
Accuracy	71.9% (64.3%–78.8%)	73.5% (63.8%–81.8%)	69.1% (55.2%–80.9%)
Discharge and ulcer-related symptoms only			
Sensitivity	52.4% (36.4%–68.0%)	55.6% (35.3%–74.5%)	46.7% (21.3%–73.4%)
Specificity	79.3% (70.8%–86.3%)	80.3% (69.5%–88.5%)	77.5% (61.6%–89.2%)
PPV	47.8% (36.7%–59.2%)	50.0% (36.2%–63.8%)	43.8% (26.1%–63.1%)
NPV	82.1% (76.8%–86.5%)	83.6% (76.7%–88.7%)	79.5% (70.0%–86.5%)
Accuracy	72.2% (64.5%–78.9%)	73.8% (64.2%–81.9%)	69.1% (55.2%–80.9%)

CI, confidence interval; PPV, positive predictive value; NPV, negative predictive value.

Results derived from: https://www.medcalc.org/calc/diagnostic_test.php.

### Experiences with implementation

Both clinic staff and patients reported positive experiences with point-of-care NAAT testing for STI diagnostics. Clinic staff viewed the incorporation of this technology as a positive addition to the clinic's services and felt it was important to continue. In particular, they mentioned it allowed them to identify and treat asymptomatic cases that may have otherwise been missed. They also reported that patients recognized the benefit of this new testing and seemed to be more active in their health management:

“On the streets people always say that we are the only organization that does those types of test that they don’t know if they can do it in any other place. And they do ask a lot about the tests and if they can come in here to have them, or some girl always passes by saying ‘Oh, I had it done, but it was negative and after 3 months I want to have it done again’, so they are always interested in monitoring their health”.—Clinic staff

Patients reported valuing the addition of a broader spectrum of STI testing than was previously available. They particularly appreciated receiving their test results faster with the point-of-care NAAT testing than with previous off-site laboratory diagnostics. Patients’ feedback on NAAT testing also incorporated overall service delivery, seeing the clinic a trustworthy service and subsequently recommended the clinic's services to their friends:

“I think is really good, the way they treat me, the test and that they gave me right away the results”.—Patient

“I told him [my partner] ‘I’m going to take you to MSF and they will make you a lot of tests’”.—Patient

“I’m thankful for the services, I’ve felt comfortable and without any judgement. When you go to a public service and you say ‘I’m here for these tests’, you are stigmatized … In [the MSF clinic] I feel confident”.—Patient

If point-of-care results were not available during the patient's clinic visit, they still had to return for an in-person consultation. In these scenarios, patients requested results to be delivered via electronic messaging, although both patients and staff raised concerns about patient privacy and delivering results without simultaneous counseling. During interviews, one patient mentioned that strengthening counseling on test results is key to ensure patients are able to make informed decisions on their health:

“I think that counseling is super important, but you have to take in consideration the knowledge that persons have when sharing information of a sensitive topic”.—Patient

Health staff noted this as a challenge, particularly with the expansion of the number of tests and consequently results to explain to the patient in a short consultation:

“They didn’t remember, [even] though we have delivered all results because we told them: ‘You are positive or negative’  … in a consultation that last 5 or 10 min, you will tell them so much that they will only keep 5% [of information]”.—Clinic staff

The primary concern raised by staff was long-term sustainability, especially with the cost of supplies and the expectation of continuous availability of these types of tests among patients:

“We were the ones that invited them [patients] to be involved. So what do people think? ‘I have a problem; they [the clinic] are going to solve it’ … when people come here and it turns out we don’t have it [the test] and we don’t notice, we are offering something we are not going to be able to provide when people come”.—Clinic staff

## Discussion

Our results highlight the high prevalence of STIs among key populations in San Pedro Sula and the limited diagnostic ability of syndromic identification in comparison to NAAT testing for STIs. While MSF's San Pedro Sula clinic focuses on sexual and reproductive health for LGBTQIA+ and sex working populations, the clinic cohort experienced a higher rate of STIs than what would be expected in a similar population in Honduras (7, 24–26) The prevalence of chlamydia, gonorrhea, syphilis, and HIV were all significantly larger than what was expected for key populations according to previous STI studies in Honduras (7, 25, 27). These findings align with those of other regional studies showing higher rates of HIV and other STIs among key populations in Honduras (28) and across Latin America (29, 30). The absence of HCV and HBV in our study population align with other studies reporting low incidences of HCV and HBV in Central America (31–33), where the overall prevalence of chronic HCV (0.73%) and HBV (0.33%) is considerably lower than the global average for HCV (2.5%) and HBV (3.2%) (34–36). These findings may be influenced by low rates of injection drug use (HCV) and higher probability of vaccination (HBV) within the MSF clinic patient population.

Risk behaviors shown in our results, such as infrequent condom use, substance use, and multiple sexual partners, coupled with high rates of STIs, could potentially contribute to the transmission of STIs both within and externally of the LGBTQIA+ and sex worker community, especially for individuals in concurrent sexual partnerships (sexual partnerships that overlap in time) or individuals with several long-term relationships at the same time (37, 38). If individuals from our study cohort who display STI risk factors engage in concurrent sexual partnerships or multiple simultaneous long-term relationships acquire an STI, they would likely expose their other partners to infection. Concurrent sexual partners increase the possibility of transmitting STIs to partners, which in turn can promote STI epidemics in populations with high concurrency, such as populations of men who have sex with men or transgender individuals (39). It is possible that the high rates of STIs in our population, such as syphilis and gonorrhea, are caused by a simultaneous combination of a lack of STI prevention behaviors (such as condom use) and concurrent sexual partnerships.

Facing an increased risk and the barriers to care, when key populations do come to health facilities for SRH care, it is even more critical to ensure STIs are correctly identified and managed. Our results suggest that the use of NAAT testing for STI screening can complement the syndromic identification of STIs in the LGBTQIA+ and sex worker communities of Honduras. Without molecular diagnostic tools, cases where symptoms are incorrectly classified as an STI (i.e: “false positive”) could contribute to the growing problem of antibiotic-resistant STIs in Latin America when prescribed antibiotic treatment incorrectly (17). Furthermore, not correctly identifying and managing an STI can contribute to acute morbidity (e.g., symptom exacerbation), long-term complications, and continued transmission. Our results suggest that NAAT testing contributes to improved diagnostics and management of STIs among key populations in San Pedro Sula, and that it is well-accepted by both staff and patients. Additionally, our findings show high rates of asymptomatic cases in the study population, which would have never been identified via syndromic management. WHO's STI management guidelines have long recommended regular asymptomatic screening of syphilis and HIV among pregnant women and key populations (40). While some literature has questioned the added value of active asymptomatic STI screening (41, 42), in 2025, WHO updated these guidelines to also conditionally recommend regular asymptomatic screening of N. gonorrhoeae and C. trachomatis among high risk populations (pregnant women and sexually active adolescents and young people in high prevalence areas; sex workers; and MSM) with treatment following national guidelines (40).

While our results highlight the added value of NAAT testing in this context, many factors can prevent the adoption and scale-up of these types of tests (43). For example, in our study, the high cost of cartridges was the most frequent concern among health staff when discussing long-time availability of NAAT testing within the clinic. The cost of supplies and equipment is a chief barrier to long-term sustainability of point-of-care NAAT testing in low- and middle-income countries like Honduras (43, 44). Efforts to reduce associated costs and research into lower-cost alternatives are needed (45).

Critically, the existence of improved diagnostic technologies does not ensure patient attendance and usage. The delivery of non-judgmental care was a cross-cutting theme in interviews and focus groups, with patients emphasizing it as a key motivator for them to attend the clinic and receive STI testing. When recommending the clinic's STI testing to friends, they often cited not only the spectrum and speed of tests, but the non-judgmental environment. In the push for better access to more advanced STI diagnostics, facilities cannot ignore the foundation on which those services stand—affirming, patient-centered care that reduces stigma and barriers.

There are several limitations to this study. First, participants were recruited from a clinic that primarily provides sexual and reproductive health services, which introduces selection bias. While this could result in higher STI rates, participants were recruited from all SRH services, not just those looking for STI testing; additionally, individuals inclined to seek SRH services may also be more likely to practice protective behaviors, which could lead to lower STI rates. Excluding patients without a working phone number also introduced selection bias. Such patients may have been economically disadvantaged and at higher risk of STIs (46, 47). This study also only investigated the most common STIs and did not test for mycoplasma, which was shown in Eswatini to be a common but often misdiagnosed co-infection (48); it is possible the overall prevalence of all STIs is higher in our population. HPV was only tested in women who accepted cervicovaginal sample (36/55; 65.5%), and test results are underpowered to detect the prevalence of HPV with a minimum of 80% power and should be considered with caution.

## Conclusion

This study adds important evidence highlighting the current prevalence of STIs in the LGBTQIA+ and sex worker community in Honduras and the feasibility of point-of-care NAAT testing at the clinic-level. Limitations of current STI screening methodologies in San Pedro Sula are a barrier to providing high-quality STI services. However, this study demonstrates that the integration of point-of-care NAAT testing into STI screening facilities provides opportunities for improved diagnostic capabilities, especially for asymptomatic cases. The LGBTQIA+ and sex worker community in San Pedro Sula face a higher burden of STIs compared to the general population and providing better STI screening through the incorporation of point-of-care NAAT testing could improve the diagnostic capabilities of healthcare services and thus improve quality of care for key populations.

## Data Availability

The raw data supporting the conclusions of this article will be made available by the authors, without undue reservation.
