# Supplementary material for: STI prevalence and the integration of point-of-care nucleic acid amplification testing into STI diagnostic algorithms at a Médecins Sans Frontières key population clinic in San Pedro Sula, Honduras
Source: Front Reprod Health. 2026 Feb 23;8:1685453. doi: 10.3389/frph.2026.1685453 (PMC12968246; doi:10.3389/frph.2026.1685453)
Supplement: Supplementary file 1 [file Presentation1.pdf]

La prevalencia de las ITS y la integración de las pruebas de amplificación de ácidos nucleicos (NAAT) en el punto de atención en los algoritmos de diagnóstico de las ITS en la clínica de población clave de MSF en San Pedro Sula, Honduras

Derek C. Johnson[1], Kimberly Rodriguez[2], Diana Gómez-López[1], Darío Rodríguez[1], Diana Dávila[2], Lindsay Salem-Bango[1+], Joaquim Guinart Verdaguer[2], Carina Perotti[2], Reinaldo Ortuño[1], Nelly Staderini,[3] Iza Ciglenecki[3]

[1] Médecins sans Frontières, Oficina Integrada de Centroamérica y México, Ciudad de México, México

[2] Médecins sans Frontières, Proyecto HN142, San Pedro Sula, Honduras

[3] Médecins Sans Frontières, Sede del Centro Operacional de Ginebra, Ginebra, Suiza

+ Autor Correspondiente: msfch-mexico-epidemiomanager@geneva.msf.org

Palabras clave: ITS, Honduras, Centroamérica, LGBTQIA+, Trabajo Sexual, NAAT, GeneXpert

S1 Appendix. Versión en español.

Resumen

Antecedentes: Los datos sobre la prevalencia de infecciones de transmisión sexual (ITS) entre poblaciones clave en Honduras son limitados. Además, los sitios de atención dependen en gran medida del manejo sindrómico de ITS, lo que tiene un bajo rendimiento diagnóstico. Este estudio evalúa la prevalencia de las ITS y la viabilidad y utilidad diagnóstica de las pruebas rápidas de amplificación de ácidos nucleicos (NAAT) en comparación con la identificación sindrómica entre la comunidad LGBTQIA+ y trabajadoras sexuales de San Pedro Sula que asisten a una clínica de Médicos sin Fronteras (MSF).

Métodos: Se invitó a participar en el estudio a los pacientes que asistieron a la clínica de MSF en San Pedro Sula entre febrero y junio de 2024. El personal clínico evaluó los síntomas de las ITS de todos los participantes y, independientemente de los síntomas, recolectaron muestras de sangre completa, orina y vagina. Se realizaron pruebas rápidas de Virus de Inmunodeficiencia Humana (VIH), Hepatitis B (VHB), Hepatitis C (VHC), sífilis y pruebas de amplificación de ácidos nucleicos (NAAT, por sus siglas en inglés) a través de GeneXpert (clamidia, gonorrea, tricomoniasis y Virus del Papiloma Humano [VPH]). El tratamiento se prescribió inicialmente según las Directrices de Manejo Sindrómico de la OMS y se revisó después de los resultados de las pruebas NAAT. Se calcularon estadísticas descriptivas y métricas diagnósticas. Se evaluó la viabilidad mediante grupos focales con el personal.

Resultados: De los 157 pacientes incluidos, el 31,8% (n=50) dieron positivo en la prueba de al menos una ITS: VPH 19,4% (7/36), sífilis 12,1% (19/157), clamidia 10,2% (16/157), gonorrea 8,3% (13/157), tricomoniasis 3,8% (6/157), VIH 3,8% (6/157), VHB 0% (0/157), y VHC 0% (0/157). De esos 38,0% (n=19) dieron positivo en la prueba de más de una ITS. Solo 49.3% de todos los participantes y 56,6% (n=22) de los que dieron positivo en la prueba de clamidia, gonorrea, sífilis o tricomoniasis eran sintomáticos. El personal consideró que las pruebas NAAT al punto de atención resultaba beneficioso para la atención de los pacientes, pero les preocupaba la sostenibilidad.

Conclusión: Este estudio destaca la prevalencia de ITS entre poblaciones clave en San Pedro Sula, Honduras. Los resultados muestran que la implementación de las pruebas NAAT es viable, útil, y deseado en el diagnóstico clínico básico. La mayor capacidad de prueba mejoró la capacidad de diagnóstico y manejo de la clínica, especialmente en lo que respecta a las ITS asintomáticas y, por consiguiente, mejoró la calidad de la atención para las poblaciones clave. Esta versión en español fue traducida del original en inglés.

Introducción

Según la Organización Mundial de la Salud (OMS), cada día se contraen más de un millón de infecciones de transmisión sexual (ITS) curables, incluyendo clamidia, gonorrea, tricomonas y sífilis[1]. Las tasas de ITS son usualmente más altas entre poblaciones clave, quienes frecuentemente enfrentan discriminación y tienen menos acceso a los servicios de salud[2]. En Honduras, las personas trabajadoras sexuales y aquellas que se identifican como lesbiana, gay, bisexual, transgénero, queer, intersexual, asexual o con otra orientación o identidad sexual (población LGBTQIA+) enfrentan estigmatización y discriminación considerables, lo que impacta en su acceso a servicios de salud sexual y reproductiva (SSR)[3,4]. Cada vez existen más acciones del gobierno para estandarizar los servicios de atención a ITS, lo que contribuirá a reducir la transmisión de ITS y la resistencia a los antimicrobianos, especialmente para poblaciones el alto riesgo de adquirirlas[5]. Para reducir la transmisión de ITS y la resistencia a los antimicrobianos, el gobierno local está haciendo cada vez más esfuerzos para estandarizar los servicios de ITS, especialmente para las poblaciones de alto riesgo. En este contexto, en 2021 Médecins sans Frontières abrió una clínica enfocada en personas LGBTQIA+ y personas trabajadoras sexuales en San Pedro Sula. La clínica brinda servicios de salud mental, sexual y reproductiva, además de vinculaciones con servicios sociales. Un equipo de promoción de la salud también realiza actividades de educación sanitaria y participación comunitaria.

Los datos sobre la prevalencia actual de ITS entre poblaciones clave en Honduras son limitados. A nivel nacional, los datos sobre ITS no se encuentran desagregados por orientación sexual, identidad de género ni condición de trabajador(a) sexual, lo que limita la disponibilidad de información pública detallada. Un estudio de 2010 sobre ITS entre hombres que tienen sexo con hombres (HSH) en Honduras documentó que 37.6% de ellos reportaron una ITS en el último año[6], y un estudio de 2006-2008 entre trabajadoras sexuales reportó una prevalencia de sífilis y clamidia de 2.3% y 6.1% respectivamente[7]. Sin embargo, esta literatura publicada tiene más de 15 años de antigüedad. Esta brecha en la investigación actual sobre la prevalencia de ITS dificulta la programación y prestación de servicios adecuada para esta población.

La prestación de servicios de ITS en Honduras también se ve obstaculizada por la limitada capacidad de diagnóstico molecular. Las clínicas en Honduras dependen en gran medida del manejo sindrómico de las ITS, o del diagnóstico y tratamiento de posibles ITS sólo a través de los síntomas[5]. Si bien las clínicas pueden enviar muestras a laboratorios regionales para realizar pruebas moleculares, los desafíos logísticos como los suministros limitados y las demoras en el transporte significan que el diagnóstico y el tratamiento iniciales se basen únicamente en los síntomas, siguiendo sólo las pautas de manejo sintomático de la OMS[8]. La OMS recomienda las pruebas moleculares, como la prueba de

amplificación de ácidos nucleicos (NAAT) – considerada ampliamente el estándar de oro para la detección de ITS[9] – para la detección y el tratamiento adecuados de las ITS, pero también proporciona pautas de manejo sindrómico para respaldar el diagnóstico y el tratamiento cuando las pruebas moleculares no están disponibles[8]. Si bien esto es útil en entornos de bajos recursos, se ha demostrado consistentemente que el manejo sindrómico tiene baja sensibilidad y especificidad. lo que puede resultar en diagnósticos erróneos y tratamientos no adecuados[9,10]. Aunque las proporciones exactas varían según la ITS y el contexto[11], la OMS reconoce que la mayoría de los casos de ITS a nivel mundial son asintomáticos[12]. Por ejemplo, algunos estudios han reportado que 58.6-95.8% (mediana 75.0%) y 13.3-99-3% (mediana 65.0%) de los casos de *N. gonorrhoeae* y de *C. tracjhomatis* respectivamente, son asintomáticos[11]. Los casos que sí son sintomáticos usualmente no tienen síntomas específicos[12]. El diagnóstico erróneo de ITS puede causar complicaciones de infecciones no tratadas como (pero no limitadas a): bajo peso al nacer, parto prematuro, aumento de la infecciosidad y la susceptibilidad al VIH y su susceptibilidad, cáncer de cuello uterino (mujeres), enfermedad inflamatoria pélvica, infertilidad, conjuntivitis neonatal y artritis reactiva[13–15]. Más aun, el diagnóstico erróneo puede conducir al uso innecesario o incorrecto de antibióticos, que puede a su vez contribuir a la resistencia de las ITS a estos antibióticos[16,17].

Los avances en las pruebas moleculares ayudan al diagnóstico y tratamiento preciso de las ITS. GeneXpert (Cepheid, Sunnyvale, CA), es una prueba diagnóstica en el punto de atención basada en la reacción en cadena de la polimerasa (PCR) en tiempo real NAAT, y se ha identificado como una forma de mejorar el diagnóstico y el tratamiento de las ITS. Estudios de viabilidad anteriores muestran que los ensayos GeneXpert se utilizan en una variedad de entornos[18–20] con más del 95% de concordancia en comparación con los métodos de PCR convencionales[21].

Para entender la prevalencia de ITS entre poblaciones clave en Honduras y la viabilidad de implementar GeneXpert para mejorar la capacidad diagnóstica, se implementó un estudio transversal en la clínica de MSF en San Pedro Sula.

Metodología

Entorno del estudio

Este estudio transversal se realizó en la clínica de MSF en San Pedro Sula, Honduras, del 19 de febrero al 3 de junio de 2024. La clínica se especializa en la provisión de servicios de salud libres de estigma para las personas LGBTQIA+

y personas trabajadoras del sexo; entre el staff se encuentra personal de enfermería, de medicina, de psicología, de trabajo social y de promoción de la salud. Provee primariamente servicios de salud mental, sexual y reproductiva, aunque algunas necesidades generales de salud son también atendidas. A los pacientes de primera vez que no son parte de la población diana de la clínica se les provee los servicios y se les vincula a otras instituciones. Antes del estudio, sólo se realizaban en el sitio pruebas rápidas de VIH y sífilis. Las muestras para las pruebas moleculares eran enviadas fuera del sitio al laboratorio regional administrado por el gobierno.

Participantes

Las personas que asistieron a la clínica de MSF en San Pedro Sula durante el período del estudio fueron evaluadas para su elegibilidad según los criterios de inclusión y exclusión. Si la persona tenía al menos 18 años, había tenido actividad sexual en los últimos 3 meses y tenía un teléfono de contacto vigente, era abordada por el equipo de MSF que explicaba el estudio y asistía en el proceso de consentimiento informado. Sólo aquellos que proveyeron consentimiento informado fueron incluidos en el estudio. Para calcular el tamaño de la muestra, las prevalencias estimadas de clamidia, gonorrea, VPH, VIH, VHB, VHC, sífilis y tricomonas fueron obtenidas de la literatura mencionada[6,7]. El tamaño de la muestra fue calculado con un 4% de precisión con el 95% de confianza. Los resultados de los tamaños de la muestra variaron según la ITS y fueron de 57 a 316 participantes: gonorrea (n=57); VHC (n=61); VIH (n=64); sífilis (n=67); VHB (n=88); tricomoniasis (n=130); clamidia (n=145); y VPH (n=316).

Para los procedimientos cualitativos, los pacientes que aceptaron participar en el estudio fueron invitados a participar en una entrevista y se recolectó un segundo consentimiento informado. El equipo de la Clínica fue invitado a participar en un grupo de discusión focal y se recolectó el consentimiento informado de aquellos que aceptaron participar.

Un diagrama de flujo del proceso del estudio puede ser encontrado en el Apéndice S2.

Procedimiento

Cada participante, independientemente de los síntomas informados, fue evaluado por el personal de enfermería y/o médico de la clínica para detectar ITS utilizando el enfoque de manejo sindrómico de la OMS. Los signos y síntomas evaluados incluyeron: disuria, dispareunia, flujo vaginal, úlcera genital, dolor genital o úlcera anal. Independientemente de los resultados de la evaluación sindrómica, cada participante se sometió a pruebas rápidas para VIH, Hepatitis B (VHB), hepatitis C (VHC), y sífilis. Las pruebas NAAT se realizó a través de GeneXpert para

genotipos de VPH de alto riesgo, clamidia, gonorrea y tricomoniasis. Para la prueba NAAT, se recolectaron muestras de orina sin diluir para clamidia, gonorrea y tricomoniasis; las muestras cervicovaginales para la prueba de VPH (para las participantes mujeres) se obtuvieron por cuenta propia o por un proveedor de atención médica, según la preferencia de la paciente. Las muestras de orina que no se procesaron dentro de las primeras 4 horas posteriores a la toma de la muestra se conservaron utilizando el kit de recolección de medio de transporte de orina CT/NG URINE-50. Las muestras para la prueba NAAT se procesaron en el sistema GeneXpert Cepheid 10-color Dx IV utilizando el software GeneXpert versión 4.3. El sistema automatiza e integra la purificación de muestras, la amplificación de ácidos nucleicos y la detección de las secuencias objetivo en las muestras mediante ensayos de PCR en tiempo real. Cepheid, el fabricante del sistema GeneXpert, ha desarrollado cartuchos para la detección de 13 tipos de VPH de alto riesgo, T. vaginalis y otros patógenos a partir de muestras de orina o hisopados, con resultados en 90 minutos[22]. El sistema GeneXpert estaba localizado en la clínica, con una fuente estable de energía para operar. Un técnico de laboratorio entrenado supervisó la carga de muestras, el mantenimiento del sistema y la entrega de resultados al equipo clínico.

Se recolectaron 5 ml de sangre completa para la prueba rápida de VIH, VHB, VHC y sífilis. Los pacientes que recibieron un resultado positivo en la prueba rápida recibieron una segunda prueba rápida durante la cita de seguimiento.

El tratamiento inicial de las ITS se proporcionó siguiendo un enfoque sindrómico, con medicación prescrita según los lineamientos de MSF[23]. Posteriormente, se actualizaba el tratamiento según fuera necesario tras la prueba NAAT. Los resultados solían estar disponibles 1-2 horas después de la consulta inicial; si la muestra se recogía al final de la jornada laboral o de la semana, los resultados estaban disponibles el siguiente día laboral. Se ofreció a los participantes la opción de esperar en la clínica a recibir los resultados o de volver más tarde ese mismo día o al día siguiente para obtenerlos. Si un participante no acudía a la clínica, se le contactaba por teléfono y se le recordaba que debía volver para recoger los resultados. Los pacientes que recibieron tratamiento para ITS bacterianas debían acudir a una visita de seguimiento a los 3 meses para volver a realizarse las pruebas, y a los 6 meses si las pruebas de sífilis resultaban positivas. Se solicitaron pruebas no treponémicas adicionales en un laboratorio externo para los pacientes con una prueba de sífilis positiva actual y antecedentes de infección previa por sífilis. A los pacientes que se pusieron en contacto con la clínica por síntomas persistentes una semana después del tratamiento antibiótico se les pidió que volvieran para una evaluación adicional.

Procedimientos cualitativos

Para evaluar la viabilidad, un grupo de discusión focal (GDF) con personal de la clínica implementadora (n=8) fue realizado un mes antes de finalizar con el estudio. Las preguntas se centraron en sus experiencias de integración de NAAT en el punto de atención en el esquema de pruebas de ITS de la clínica. Para incluir la perspectiva de pacientes, se realizaron 26 entrevistas entre quienes aceptaron participar en el estudio. Las entrevistas fueron realizadas en persona o vía telefónica según la preferencia y disponibilidad de los participantes. Las preguntas se centraron en sus perspectivas sobre la atención prestada por MSF, la disponibilidad de las pruebas y las necesidades de salud sexual. Para mitigar los sesgos en las respuestas, las entrevistas fueron realizadas por miembros del equipo de investigación que no estaban en contacto directo con los pacientes, y el GDF fue realizado por una investigadora que no era parte del equipo de la clínica. Ambos, el GDF y las entrevistas, fueron realizados en español, los testimonios incluidos en la versión en inglés fueron traducidos ex profeso para ese artículo. Tanto en el grupo de discusión como en las entrevistas, se realizaron análisis temáticos para identificar los temas clave de las respuestas.

Análisis Estadístico

Las ITS se clasificaron en dos categorías para el análisis basado en las directrices de manejo sindrómico de la OMS[8], para su análisis: 1) "Cualquier ITS", que incluye todas las ITS examinadas en el estudio, y 2) "ITS tratadas con antibióticos", que incluyen clamidia, gonorrea, tricomoniasis y sífilis. Se utilizaron estadísticas descriptivas y tablas de resumen para describir a la población participante y los resultados de las pruebas de ITS. Los indicadores para determinar la prevalencia de ITS se basaron en el número de pruebas NAAT positivas y negativas para cada ITS. Cuando correspondía, se evaluó la significancia estadística mediante pruebas univariadas. Para las pruebas univariadas, las pruebas de Chi-cuadrada de Pearson y la prueba exacta de Fisher para evaluar la significancia estadística entre variables categóricas. Para las pruebas univariadas que evalúan la significancia entre variables categóricas y continuas se usó la prueba de Suma de Rangos de Wilcoxon. Se determinó la sensibilidad/especificidad y el valor predictivo positivo (VPP)/valor predictivo negativo (VPN) de las pruebas sindrómicas para el resultado positivo de una o más de las siguientes ITS: 1) clamidia, 2) gonorrea, 3) sífilis, 4) tricomoniasis, donde el resultado positivo de una ITS mediante la prueba NAAT se consideró un "valor verdadero" estándar de oro para la positividad de ITS. Se analizaron las diferencias entre el número de casos de ITS identificados correctamente mediante métodos sindrómicos en comparación con la prueba NAAT según el sexo asignado al nacer.

Resultados

Características descriptivas

Entre el 19 de febrero y el 3 de junio de 2024, se inscribieron en el estudio 157 personas. La mediana de edad fue de 29 años (Rango Intercuartílico [(IQR]) 23 – 33 años) y el 65.0% (n=102) eran varones biológicos (Tabla 1). La identidad de género más común fue la de hombre cisgénero (59.9%, n=94), seguida de la de mujer cisgénero (36.3%, n=57). Las mujeres transgénero y las personas no binarias representaron el 3.1% (n=5) de la población. Hubo una diferencia significativa en la orientación sexual entre hombres con sexo masculino asignado al nacer y mujeres con sexo femenino asignado al nacer, donde la mayoría de los hombres se identificaron como homosexuales (72.5%, n=74) y la mayoría de las mujeres se identificaron como heterosexuales (63.6%, n = 35; valor $p < 0.001$). La mayoría de los participantes del estudio eran solteros (74.5%, n=118) o vivían con su pareja, pero no estaban casados (20.4%, n=32). Hubo una diferencia global significativa en el estado civil y el sexo biológico, con más hombres que mujeres que informaron ser solteros (82.0%, n=84 frente a 60.0%, n=33) y más mujeres que hombres vivían con su pareja, pero no estaban casadas (30.9%, n=17 frente a 15.0%, n=15). El 26.8% de los participantes (n=42) informaron haber sido diagnosticados previamente con una ITS. Los varones biológicos tenían más del doble de probabilidades de informar una ITS previa que las mujeres (33.3%, n=34 frente a 14.5%, n=8; $p<0.05$) (Tabla 1).

Prevalencia de ITS

El 31.8% de los participantes (n=50) dieron positivo para al menos una ITS. Los porcentajes de ITS basados en la población total como denominador fueron los siguientes: VPH 19.4% (n=7), sífilis 12.1% (n=19), clamidia 10.2% (n=16), gonorrea 8.3% (n=13), tricomoniasis 3.8% (n=6), VIH 3.8% (n=6), VHB 0% (n=0), y VHC 0% (n=0) (Tabla 2). De los diagnosticados con una ITS, el 38% (n=19) dieron positivo en la prueba de más de una ITS La tricomoniasis era la más probable que en ser acompañada de una coinfección (100.0% n=6 de las infecciones por tricomoniasis), seguida del VIH (66.6%, n=4), clamidia (56.3%, n=9), gonorrea (53.8%, n=7), sífilis (52.6%, n=10), VPH (28.6%, n=2) (Tabla 3)

Tabla 1: Características descriptivas de los participantes, estratificados por sexo y diagnóstico de ITS

	**Todo** (n=157)	**Sexo** Masculino (n=102)	Femenino (n=55)	Valor de p[4]	**Diagnóstico de ITS** Ninguno (n=107)	Cualquier ITS (n=50)	ITS Tratada con Antibióticos[5] (n=41)	Valor de p[4]
**Sociodemográficos**[1]								
**Edad**[1]	29 (8)	29 (8)	29 (8)	0.7	28 (24, 33)	27 (21, 33)	26 (21, 33)	0.3
**Sexo**[3,7]								
Masculino	102 (65.0%)	--	--		73 (68.2%)	29 (58.0%)	26 (63.1%)	0.3
Femenino	54 (35.0%)	--	--		34 (31.8%)	20 (40.0%)	15 (36.6%)	
**Identidad de género**[3, 6]				**<0.001*** **				0.6
Hombre cisgénero	94 (59.9%)	94 (92.1%)	0 (0.0%)		67 (62.6%)	27 (54.0%)	23 (56.1%)	
Mujer cisgénero	57 (36.3%)	2 (2.0%)	55 (100.0%)		37 (34.6%)	20 (40.0%)	15 (36.6%)	
Mujer transgénero	4 (2.5%)	4 (3.9%)	0 (0.0%)		2 (1.9%)	2 (4.0%)	2 (4.9%)	
No binarie	1 (0.6%)	1 (1.0%)	0 (0.0%)		1 (0.9%)	0 (0.0%)	0 (0.0%)	
**Orientación sexual**[3,6]				**<0.001*** **				0.11
Heterosexual	37 (23.6%)	2 (2.0%)	35 (63.6%)		20 (18.7%)	17 (34.0%)	7 (17.1%)	
Homosexual	74 (47.1%)	74 (72.5%)	0 (0.0%)		55 (51.4%)	19 (38.0%)	16 (39.0%)	
Lesbiana	9 (5.7%)	1 (1.0%)	8 (14.5%)		7 (6.5%)	2 (4.0%)	14 (34.1%)	
Bisexual	30 (19.7%)	19 (18.6%)	12 (21.8%)		22 (20.6%)	8 (16.0%)	1 (2.4%)	
Otro	6 (3.8%)	6 (5.9%)	0 (0.0%)		3 (2.8%)	3 (6.0%)	3 (7.3%)	
**Estado marital**[3]				**0.009** **				0.3
Casado	6 (3.8%)	2 (2.0%)	4 (7.3%)		4 (3.7%)	2 (4.0%)	2 (4.9%)	
Divorciado	1 (0.6%)	1 (1.0%)	0 (0.0%)		1 (0.9%)	0 (0.0%)	0 (0.0%)	
Soltero	118 (74.5%)	84 (82.4%)	33 (60.0%)		84 (78.5%)	34 (68.0%)	29 (70.7%)	
Vive con pareja (no casado)	32 (20.4%)	15 (14.7%)	17 (30.9%)		18 (16.8%)	14 (28.0%)	10 (24.4%)	
**Hijos (Sí)** [3]	43 (27.3%)	8 (7.8%)	35 (63.6%)	**<0.001*** **	30 (28.0%)	13 (26.0%)	11 (26.8%)	0.56
**Historial de ITS**[3]								
**Pruebas de ITS previas**	132 (84.1%)	88 (86.3%)	44 (80.0%)	0.4	91 (85.0.%)	41 (82.0%)	34 (82.9%)	0.09
**Diagnósticos de ITS previos**	42 (26.8%)	34 (33.3%)	8 (14.5%)	**0.013***	22 (20.6%)	20 (40.0%)	17 (41.1%)	**0.005** **
**Factores de riesgo**								

**Pareja con diagnóstico de ITS en los últimos 3 meses (Sí)** [3]	21 (13.4%)	17 (16.7%)	4 (7.3%)	0.095	15 (14.0%)	6 (12.0%)	5 (12.2%)	0.7
**Núm. de parejas sexuales en el último mes** [2]	1 (1, 4)	1 (1, 3)	2 (1, 9)	**0.017***	1 (1, 3)	1 (1, 4)	1 (1, 5)	0.4
**Uso actual de drogas** [3]				0.3				
Cualquier tipo	33 (21.0%)	24 (23.5%)	9 (16.4%)		23 (21.5%	10 (20.0%)	7 (17.1%)	
Inyectable	3 (1.9%)	2 (2.0%)	1 (1.8%)		2 (1.9%)	1 (2.0%)	0 (0.0%)	
**Habla con sus parejas sobre prevención de ITS** [3]	101 (64.3%)	69 (67.6%)	32 (58.2%)	0.2	75 (70.1%)	27 (54.0%)	22 (53.7%)	**0.046***
**Frecuencia de uso de condón** [3]				0.5				0.2
Siempre	43 (26.8%)	33 (32.4%)	10 (18.2%)		33 (30.8%)	10 (20.0%)	9 (22.0%)	
Regularmente	13 (8.3%)	8 (7.8%)	5 (9.1%)		10 (9.3%)	3 (6.0%)	3 (7.3%)	
A veces	40 (25.5%)	26 (25.5%)	14 (25.5%)		28 (26.2%)	12 (24.0%)	9 (22.0%)	
Casi nunca	2 (1.3%)	2 (2.0%)	0 (0.0%)		0 (0.0%)	2 (4.0%)	2 (4.9%)	
Nunca	4 (2.5%)	2 (2.0%)	2 (3.6%)		2 (1.9%)	2 (4.0%)	2 (4.9%)	

[1] Faltaba información demográfica de 1 individuo
[2] Mediana (IQR).
[3] n (%).
[4] Prueba de suma de rangos de Wilcoxon; Prueba exacta de Fisher; Prueba de chi-cuadrada de Pearson.
[5] Clamidia, gonorrea, tricomoniasis y sífilis.
[6] La identidad de género y la orientación sexual fueron auto reportadas
[7] Para la comparación estadística de “Sexo” y “Cualquier ITS” y “ITS tratado con antibiótico”, sólo se incluyeron clamidia, gonorrea y sífilis en el análisis.

Tabla 2. Diagnóstico de ITS

	Cualquier ITS	Ninguna ITS	Clamidia	Gonorrea	Sífilis	Tricomoniasis	VIH	VPH[&]	VBH	VCH
**Positivo[1,3]**	50 (31.8%)	107 (68.2%)	16 (10.2%)	13 (8.3%)	19 (12.1%)	6 (3.8%)	6 (3.8%)	7 (19.4%)	0 (0.0%)	0 (0.0%)
**Sexo[3,4]**										
Masculino	29 (58.0%)	73 (68.2%)	5 (31.3%)**	10 (76.9%)	17 (89.5%)*	--	5 (83.3%)	--	0 (0.0%)	0 (0.0%)
Femenino	20 (40.0%)	34 (31.8%)	11 (68.8%)	2 (15.4%)	2 (10.5%)	6 (100%)	1 (16.7%)	7 (19.4%)	0 (0.0%)	0 (0.0%)
**Síntomas[2,3]**										
Cualquiera	23 (46.0%)	23 (21.5%)	7 (43.8%)	12 (92.3%)	10 (52.6%)	1 (16.7%)	3 (50.0%)	0 (0.0%)	0 (0.0%)	0 (0.0%)
Disuria	11 (22.0%)	7 (6.5%)	1 (6.3%)	9 (69.2%)	3 (15.8%)	1 (16.7%)	3 (50%)	0 (0.0%)	0 (0.0%)	0 (0.0%)
Dispareunia	8 (16.0%)	6 (5.6%)	2 (12.6%)	4 (30.8%)	3 (15.8%)	1 (16.7%)	1 (16.7%)	0 (0.0%)	0 (0.0%)	0 (0.0%)
Secreción vaginal[5]	6 (30.0%)	5 (4.7%)	2 (12.6%)	4 (30.8%)	1 (5.3%)	0 (0.0%)	0 (0.0%)	0 (0.0%)	0 (0.0%)	0 (0.0%)
Úlceras genitales	1 (2.0%)	0 (0.0%)	0 (0.0%)	0 (0.0%)	1 (5.3%)	0 (0.0%)	1 (16.7%)	0 (0.0%)	0 (0.0%)	0 (0.0%)
Úlceras anales	0 (0.0%)	1 (0.9%)	0 (0.0%)	0 (0.0%)	0 (0.0%)	0 (0.0%)	0 (0.0%)	0 (0.0%)	0 (0.0%)	0 (0.0%)
Vesícula genital / ampolla	0 (0.0%)	1 (0.9%)	0 (0.0%)	0 (0.0%)	0 (0.0%)	0 (0.0%)	0 (0.0%)	0 (0.0%)	0 (0.0%)	0 (0.0%)

[1] El porcentaje de resultados positivos se calculó utilizando el número total de participantes (157) como denominador.
[2] El porcentaje con síntomas se calculó utilizando el número de pruebas positivas para la ITS indicada como denominador.
[3] n(%).
[4] Cuando corresponde, se realizaron las Prueba de suma de rangos de Wilcoxon; Prueba exacta de Fisher; Prueba de chi-cuadrada de Pearson en diferencias por sexo.
[5] Sólo mujeres biológicas fueron consideradas.
* p<0.05, ** p<0.01, *** p<0.001.
[&] Sólo 36 mujeres recibieron detección de VPH.

Tabla 3. Número de coinfecciones de ITS

	Clamidia (N=9)	Gonorrea (N=7)	Sífilis (N=10)	Tricomoniasis (N=6)	VPH (N= 2)	VIH (N=4)
**Clamidia (N=9)**						
**Gonorrea (N=7)**	2					
**Sífilis (N=10)**	3	4				
**Tricomoniasis (N=6)**	3	0	1			
**VPH (N = 2)**	1	0	0	1		
**VIH (N=4)**	0	1	2	1	0	

Las personas que previamente habían dado positivo a una ITS tenían más del doble de probabilidades de dar positivo en la prueba durante su visita a la clínica que las personas que nunca habían dado positivo en la prueba de una ITS (40.0% frente a 20.6%) (Tabla 1). Si bien no hubo una diferencia significativa en la incidencia general de ITS según el sexo, sí hubo una diferencia significativa para ITS específicas. Significativamente más hombres (16.6%) que mujeres (3.2%) dieron positivo en la prueba de sífilis ($p<0.05$). Por el contrario, más mujeres (17.5%) dieron positivo en la prueba de clamidia que hombres (4.9 %) ($p<0,01$).

Síntomas de ITS

El 29.5% (n=46) de todos los participantes informaron síntomas de ITS, siendo la disuria la más común (39.0% de las personas que informaron síntomas, n=18), seguida de la dispareunia (30.0%, n=14) y el flujo vaginal (20.0%, n=11 de las mujeres que informaron síntomas) (Tabla 2). De los 46 participantes con síntomas de ITS, el 50.0% (n=23) posteriormente dio positivo en la prueba de ITS. Sin embargo, no todos los participantes con una ITS informaron síntomas (54.0%, N=27). De las pruebas de NAAT positivas para clamidia, gonorrea, sífilis o tricomoniasis, solo el 56.6% informó síntomas: gonorrea 92.3% (12/13), sífilis 52.6% (10/19), clamidia 43.8% (7/16), y tricomoniasis 16.7% (1/6).

Manejo Sindrómico vs. Pruebas de NAAT

La sensibilidad y especificidad de la identificación sindrómica de las ITS en comparación con el cribado mediante NAAT fue del 53.7% (IC del 95%: 37.4 – 69.3%) y del 78.5% (IC del 95%: 69.9 – 85.5%) respectivamente, con un valor predictivo positivo (VPP) del 46.8% (IC del 95%: 35.9 – 58.0%) y un valor predictivo negativo (VPN) del 82.7% (IC del 95%CI: 77.3 – 87.1%), y una precisión del 71.9% (IC del 95%: 64.3 – 78.8%) (Tabla 4). La sensibilidad y

especificidad de los síntomas específicos para las ITS, donde los síntomas de secreción se utilizaron para evaluar gonorrea, clamidia o tricomonas y las úlceras se utilizaron para evaluar la sífilis fue de 53.4% (IC del 95%: 31.8 – 60.7%) y 79.3% (IC del 95%: 70.8 – 83.6%), con un VPP de 47.8% (IC del 95%: 36.7 – 59.2%) y un VPN de 82.1% (IC del 95%: 76.8% a 66.5%) (Tabla 4). En ambos casos, los hombres tuvieron mejor sensibilidad, especificidad, VPP y VPN que las mujeres con respecto a la identificación sindrómica de las ITS.

Tabla 4. Métricas de prueba.

Métrica	Todos (95% IC)	Hombres (95% IC)	Mujeres (95% IC)
**Manejo sindrómico**			
Sensibilidad	53.7% (37.4% - 69.3%)	48.4% (30.2% - 66.9%)	46.7% (21.3% - 73.4%)
Especificidad	78.5% (69.9% - 85.5%)	84.5% (73.9% - 92.0%)	77.5% (61.6% - 89.2%)
VPP [a]	46.8% (35.9% - 58.0%)	57.7% (41.5% - 72.4%)	43.8% (26.1% - 63.1%)
VPN [a]	82.7% (77.3% - 87.1%)	78.9% (72.5% - 84.3%)	79.5% (70.1% - 86.5%)
Precisión	71.9% (64.3% - 78.8%)	73.5% (63.8% - 81.8%)	69.1% (55.2% - 80.9%)
**Solo la secreción y síntomas relacionados con la úlcera**			
Sensibilidad	52.4% (36.4% - 68.0%)	55.6% (35.3% - 74.5%)	46.7% (21.3% - 73.4%)
Especificidad	79.3% (70.8% - 86.3%)	80.3% (69.5% - 88.5%)	77.5% (61.6% - 89.2%)
VPP	47.8% (36.7% - 59.2%)	50.0% (36.2% - 63.8%)	43.8% (26.1% - 63.1%)
VPN	82.1% (76.8% - 86.5%)	83.6% (76.7% - 88.7%)	79.5% (70.0% - 86.5%)
Precisión	72.2% (64.5% - 78.9%)	73.8% (64.2% - 81.9%)	69.1% (55.2% - 80.9%)

*Resultados de: https://www.medcalc.org/calc/diagnostic_test.php
IC: Intervalo de confianza; VPP: valor predictivo positivo; VPN: valor predictivo negative

Experiencia con la implementación

Tanto el personal de la clínica como los pacientes informaron de experiencias positivas con las pruebas NAAT en el punto de atención para el diagnóstico de ITS. El personal de la clínica consideró que la incorporación de esta tecnología era una adición positiva a los servicios de la clínica y que era importante continuar con ella. En particular, mencionaron que les permitía identificar y tratar casos asintomáticos que de otro modo podrían haberse pasado por alto. También informaron de que los pacientes reconocían los beneficios de estas nuevas pruebas y parecían ser más activos en la gestión de su salud:

En la calle siempre dicen que somos la única organización que les hace ese tipo de pruebas, que no saben si se las pueden hacer en otro sitio. Y preguntan bastante por las pruebas y si se las pueden venir hacer o alguna chica

siempre pasa diciendo ' ay yo me la hice, pero me salió negativa y después de 3 meses me la quiero volver a hacer ', entonces están interesadas en darle un seguimiento a su salud." -Personal de la clínica

Los pacientes afirmaron valorar la incorporación de un espectro de pruebas de ITS más amplio que el disponible anteriormente. Apreciaron especialmente que las pruebas NAAT en el punto de atención les permitiera recibir los resultados de las pruebas con mayor rapidez que los anteriores diagnósticos de laboratorio externos. La retroalimentación general sobre las pruebas NAAT incorporó también la prestación general del servicio, percibiendo la clínica como un servicio digno de confianza y que, subsecuentemente, recomendaron a sus pares:

"*Me parece muy bien, el trato, la prueba y que me dieran enseguida los resultados."* – Paciente

"*Le dije (a mi pareja) 'te voy a llevar a MSF y te van a hacer muchas pruebas'*". – Paciente

"Agradezco los servicios, me sentí cómodo y sin ningún juicio. Cuando vas a los servicios públicos y dices 'vengo por estas pruebas', te estigmatizan… Aquí [en la clínica de MSF] *me siento confiado".* – Paciente

Si los resultados en punto de atención no estaban disponibles durante la visita clínica del paciente, éste tenía que volver para una consulta en persona. En estos casos, los pacientes solicitaron que los resultados se les enviaran por mensajería electrónica, aunque el personal expresó su preocupación por la privacidad del paciente y la entrega de los resultados sin asesoramiento simultáneo. Tanto los pacientes como el personal expresaron su preocupación por el asesoramiento. Durante las entrevistas, un paciente mencionó que reforzar la consejería sobre los resultados de las pruebas es clave para garantizar que los pacientes puedan tomar decisiones informadas sobre su salud:

"*Creo que la consejería es super importante, pero hay que tener en cuenta los conocimientos que tienen las personas a la hora de compartir información sobre un tema delicado*." – Paciente

El personal sanitario también señaló esto como un reto, sobre todo con la ampliación del número de pruebas y, en consecuencia, de resultados que explicar al paciente en una consulta breve.

"*Ellos no se acordaban [aunque] les hemos entregado todos los resultados porque se lo hemos dicho: 'Usted es positivo o negativo'... en una consulta que dura 5 o 10 minutos, les dirás tantas cosas que sólo se quedarán con el 5% [de la información]*." - Personal clínico

La principal preocupación planteada por el personal era la sostenibilidad a largo plazo, especialmente con el coste de los suministros y la expectativa de disponibilidad continua de este tipo de pruebas entre los pacientes:

"*Fuimos nosotros quienes invitaron [a los pacientes] a participar. ¿Y qué piensa la gente? 'Tengo un problema; ellos [la clínica] van a resolverlo'... cuando la gente viene aquí y resulta que no tenemos [la prueba] y no nos*

damos cuenta, estamos ofreciendo algo que no vamos a poder proveer cuando venga la gente". - Personal de la clínica

Discusión

Nuestros resultados destacan la alta prevalencia de ITS entre las poblaciones clave en San Pedro Sula y la limitada capacidad diagnóstica de la identificación sindrómica en comparación con las pruebas NAAT para ITS. Aunque la clínica de MSF en San Pedro Sula se centra en la salud sexual y reproductiva de las poblaciones LGBTQIA+ y trabajadoras sexuales, la cohorte de la clínica experimentó una tasa de ITS más alta de lo que cabría esperar en una población similar en Honduras[7,24–26]. La prevalencia de clamidia, gonorrea, sífilis y VIH fue significativamente mayor de la esperado para poblaciones clave según estudios previos de ITS en Honduras[7,25,27]. Estos hallazgos coinciden con los de otros estudios regionales que muestran tasas más altas de VIH y otras ITS entre las poblaciones clave en Honduras[28] y en toda América Latina[29,30]. La ausencia de VHC y VHB en nuestra población de estudio coincide con otros estudios que informan bajas incidencias de VHC y VHB en América Central[31–33], donde la prevalencia general de VHC crónica (0.73%) y VHB (0.33%) es considerablemente inferior a la media mundial de VHC (2.5%) y VHB (3.2%)[34–36]. Estos hallazgos pueden verse influidos por las bajas tasas de uso de drogas inyectables (VHC) y la mayor probabilidad de vacunación (VHB) dentro de la población de pacientes de las clínicas de MSF.

Los comportamientos de riesgo mostrados en nuestros resultados, como el uso infrecuente del condón, el consumo de sustancias y las múltiples parejas sexuales, junto con las altas tasas de ITS, podrían contribuir a la transmisión de ITS tanto dentro como fuera de la comunidad LGBTQIA+ y de trabajadores sexuales, especialmente en el caso de personas con parejas sexuales simultáneas (parejas sexuales que se superponen en el tiempo) o personas con varias relaciones a largo plazo al mismo tiempo[37,38]. Si los individuos de nuestra cohorte de estudio que presentan factores de riesgo de ITS mantienen relaciones sexuales simultáneas o varias relaciones de larga duración simultáneas adquieren una ITS, probablemente expondrían a sus otras parejas a la infección. Las parejas sexuales simultáneas aumentan la posibilidad de transmisión de ITS a las parejas, lo que a su vez puede promover epidemias de ITS en poblaciones con alta concurrencia, como las poblaciones de hombres que tienen relaciones sexuales con hombres o personas transgénero[39]. Es posible que las elevadas tasas de ITS en nuestra población, como la sífilis y la gonorrea, estén causadas por una

combinación simultánea de falta de conductas de prevención de las ITS (como el uso del preservativo) y la concurrencia de parejas sexuales.

Considerando el mayor riesgo y las barreras para la atención que enfrentan, cuando las poblaciones clave acuden a los centros de salud para recibir atención en SSR, es aún más crítico asegurar que las ITS sean correctamente identificadas y manejadas. Nuestros resultados sugieren que el uso de pruebas NAAT para la detección de ITS puede complementar la identificación sindrómica de ITS en las comunidades LGBTQIA+ y de trabajadoras sexuales de Honduras. Sin herramientas de diagnóstico molecular, los casos en los que los síntomas se clasifican incorrectamente como una ITS (es decir, "falso positivo") podrían contribuir al creciente problema de las ITS resistentes a los antibióticos en América Latina cuando se prescribe un tratamiento antibiótico de forma incorrecta[17]. Además, no identificar y tratar correctamente una ITS puede contribuir a la morbilidad aguda (p. ej., exacerbación de los síntomas), complicaciones a largo plazo y transmisión continua. Nuestros resultados sugieren que las pruebas NAAT contribuyen a la mejora del diagnóstico y el manejo de las ITS entre poblaciones clave en San Pedro Sula, y que son bien aceptadas tanto por el personal como por los pacientes. Además, nuestros hallazgos muestran altas tasas de casos asintomáticos en la población del estudio, que nunca se habrían identificado mediante el manejo sindrómico. Las guías de manejo de las ITS de la OMS han recomendado desde hace tiempo la realización periódica de tamizaje en personas asintomáticas para sífilis y VIH entre mujeres embarazadas y poblaciones clave[40]. Si bien parte de la literatura científica ha cuestionado el valor añadido del tamizaje activo de ITS en personas asintomáticas [41,42], en 2025 la OMS actualizó estas guías para recomendar de manera condicional el tamizaje periódico en personas asintomáticas para *Neisseria gonorrhoeae* y *Chlamydia trachomatis* en poblaciones de alto riesgo (mujeres embarazadas y adolescentes y jóvenes sexualmente activos en zonas de alta prevalencia; trabajadores/as sexuales; y hombres que tienen sexo con hombres), con tratamiento conforme a las guías nacionales[40].

Mientras que nuestros resultados resaltan el valor añadido de las pruebas NAAT en este contexto, diferentes factores pueden impactar en la adopción y escalamiento de este tipo de pruebas[43]. Por ejemplo, en nuestro estudio, el alto costo de los cartuchos fue una de las principales preocupaciones entre el personal de salud cuando se discutía la disponibilidad a largo tiempo de las pruebas NAAT dentro de la clínica. El costo de los suministros y equipo es una gran barrera para la sostenibilidad a largo plazo de las pruebas NAAT en los puntos de atención en países de bajo e ingreso medio, como Honduras[43,44]. Los esfuerzos para reducir los costos asociados y la investigación sobre alternativas menos costosas son necesarias[45].

De manera crítica, la existencia de tecnologías diagnósticas mejoradas no asegura la asistencia de los pacientes ni su uso. La atención sin prejuicios fue un tema transversal en las entrevistas y grupos focales, donde los pacientes la destacaron como un factor clave para acudir a la clínica y hacerse las pruebas de ITS. Al recomendar las pruebas de ITS de la clínica a sus amigos, a menudo mencionaban no solo la variedad y la rapidez de las pruebas, sino también el entorno sin prejuicios. En su afán por un mejor acceso a diagnósticos de ITS más avanzados, los centros no pueden ignorar la base sobre la que se asientan estos servicios: una atención afirmativa y centrada en el paciente que reduce el estigma y las barreras.

Este estudio tiene varias limitaciones. En primer lugar, los participantes fueron reclutados en una clínica que brinda principalmente servicios de salud sexual y reproductiva, lo que introduce un sesgo de selección. Si bien esto podría dar como resultado tasas más altas de ITS, los participantes fueron reclutados en todos los servicios de SSR, no solo en aquellos que buscaban pruebas de ITS; además, las personas inclinadas a buscar servicios de SSR también pueden tener más probabilidades de practicar conductas protectoras, lo que podría conducir a tasas más bajas de ITS. La exclusión de pacientes sin un número de teléfono que funcione también introdujo un sesgo de selección. Dichos pacientes pueden haber estado económicamente desfavorecidos y tener un mayor riesgo de ITS[46,47]. Este estudio también investigó únicamente las ITS más comunes y no realizó pruebas de micoplasma, que en Esuatini demostró ser una coinfección común[48]; pero a menudo mal diagnosticada es posible que la prevalencia general de todas las ITS sea mayor en nuestra población. El VPH sólo se analizó en mujeres que aceptaron la toma de la muestra cervicovaginal (36/55; 65.5%), y los resultados de las pruebas no tienen potencia suficiente para detectar la prevalencia del VPH con una potencia mínima del 80%, por lo que deben considerarse con precaución.

Conclusión

Este estudio agrega evidencia importante que destaca la prevalencia actual de las ITS en la comunidad LGBTQIA+ y de trabajadores sexuales en Honduras y el valor agregado y la viabilidad de las pruebas NAAT al punto de atención a nivel clínico. Las limitaciones de las metodologías actuales de detección de ITS en San Pedro Sula son una barrera para brindar servicios de ITS de alta calidad. Sin embargo, este estudio demuestra que la integración de las pruebas NAAT en los sitios de detección de ITS brinda oportunidades para mejorar las capacidades de diagnóstico. La comunidad LGBTQIA+ y de trabajadores sexuales en San Pedro Sula enfrenta una mayor carga de ITS en comparación con la población general y brindar una mejor detección de ITS a través de la incorporación de las pruebas

NAAT al punto de atención podría mejorar las capacidades de diagnóstico de los servicios de atención médica y mejorar la calidad de la atención para las poblaciones claves.

Contribuciones de las y los autores

DJ, JGV, IC, RO, CP y DGL contribuyeron para concebir y diseñar el estudio. KR, DR, DGL y DD contribuyeron para la recolección y sistematización de datos. DJ, LSB, DR, DGL y IC contribuyeron al análisis de los datos. DJ, LSB, DGL realizaron el análisis y escribieron el artículo. Todos los y las autores contribuyeron a revisar y editar el artículo.

Conflicto de intereses

Los autores declaran no tener ningún conflicto de intereses.

Reconocimientos

Nos gustaría agradecer a todos los pacientes que participaron en el estudio, así como a todo el personal de la clínica de MSF en San Pedro Sula, Honduras.

S2 Apéndice. Diagrama de flujo

Referencias

1. World Health Organization. Global Health Sector Strategies on, Respectively, HIV, Viral Hepatitis and Sexually Transmitted Infections for the Period 2022-2030. 1st ed. Geneva: World Health Organization, 2022.

2. Pan American Health Organization. Framework for Monitoring HIV/STI Services for Key Populations in Latin America and the Caribbean. 2019: PanAmerican Health Organization, https://iris.paho.org/handle/10665.2/51682.

3. Secretaría de Salud. Manual de procedimientos clínicos para el manejo de las infecciones de transmisión sexual (ITS), https://platform.who.int/docs/default-source/mca-documents/policy-documents/operational-guidance/HND-RH-43-03-OPERATIONAL-GUIDANCE-2014-esp-Manual-de-procedimientos-ITS.pdf (2014).

4. Polanco PM, Rivas V. El sistema de salud en Honduras ante la diversidad sexual. Rev Médica Hondureña 2022; 90: 167–168.

5. Secretaría de Salud. Manual para el manejo clínico de las infecciones de transmisión sexual, https://salud.gob.hn/sshome/index.php/component/jdownloads/?task=download.send&id=314 (2021).

6. Tinajeros F, Artiles N, Farach N, et al. P1-S2.42 STI prevalence and condom use in men who have sex with men attending STI Services, Honduras 2010. Sex Transm Infect 2011; 87: A140.2-A141.

7. Tinajeros F, Miller WM, Castro L, et al. Declining sexually transmitted infections among female sex workers: the results of an HIV and sexually transmitted infection prevention strategy in Honduras, 2006–08. Int J STD AIDS 2012; 23: 88–93.

8. World Health Organization. Guidelines for the management of symptomatic sexually transmitted infections. Geneva, Switzerland: World Health Organization, 2021.

9. Cheng Y, Paintsil E, Ghebremichael M. Syndromic versus Laboratory Diagnosis of Sexually Transmitted Infections in Men in Moshi District of Tanzania. AIDS Res Treat 2020; 2020: 1–7.

10. Wi TE, Ndowa FJ, Ferreyra C, et al. Diagnosing sexually transmitted infections in resource-constrained settings: challenges and ways forward. J Int AIDS Soc 2019; 22: e25343.

11. World Health Organization. Guidelines for the management of asymptomatic sexually transmitted infections. Web Annex: Evidence-to-decision framework and systematic review for the management of asymptomatic sexually transmitted infections. Geneva: World Health Organization. Epub ahead of print 9 July 2025. DOI: 10.2471/B09199.

12. World Health Organization. Sexually transmitted infections (STIs), https://www.who.int/news-room/fact-sheets/detail/sexually-transmitted-infections-(stis) (accessed 19 December 2025).

13. Deal C, Cates W, Peeling R, et al. Long-term Clinical Sequelae of Sexually Transmitted Infections in Women1. Emerg Infect Dis 2004; 10: e2–e2.

14. Finnegan LP, Sheffield J, Sanghvi H, et al. Infectious Diseases and Maternal Morbidity and Mortality. Emerg Infect Dis 2004; 10: e17–e17.

15. Sentís A, Martin-Sanchez M, Arando M, et al. Sexually transmitted infections in young people and factors associated with HIV coinfection: an observational study in a large city. BMJ Open 2019; 9: e027245.

16. Cristillo AD, Bristow CC, Torrone E, et al. Antimicrobial Resistance in Neisseria gonorrhoeae: Proceedings of the STAR Sexually Transmitted Infection—Clinical Trial Group Programmatic Meeting. Sex Transm Dis 2019; 46: e18–e25.

17. Sandoval MM, Bardach A, Rojas-Roque C, et al. Antimicrobial resistance of Neisseria gonorrhoeae in Latin American countries: a systematic review. J Antimicrob Chemother 2023; 78: 1322–1336.

18. Peters RPH, De Vos L, Maduna L, et al. Laboratory Validation of Xpert Chlamydia trachomatis/Neisseria gonorrhoeae and Trichomonas vaginalis Testing as Performed by Nurses at Three Primary Health Care Facilities in South Africa. J Clin Microbiol 2017; 55: 3563–3565.

19. Badman SG, Willie B, Narokobi R, et al. A diagnostic evaluation of a molecular assay used for testing and treating anorectal chlamydia and gonorrhoea infections at the point-of-care in Papua New Guinea. Clin Microbiol Infect 2019; 25: 623–627.

20. Hesse EA, Widdice LE, Patterson-Rose SA, et al. Feasibility and acceptability of point-of-care testing for sexually transmissible infections among men and women in mobile van settings. Sex Health 2015; 12: 71.

21. Causer LM, Guy RJ, Tabrizi SN, et al. Molecular test for chlamydia and gonorrhoea used at point of care in remote primary healthcare settings: a diagnostic test evaluation. Sex Transm Infect 2018; 94: 340–345.

22. GeneXpert | Sexual Health, http://www.cepheid.com/en/cepheid-solutions/clinical-ivd-tests/sexual-health.

23. Mèdecins sans Frontieres. Major genital infections - MSF Medical Guidelines, https://medicalguidelines.msf.org/en/viewport/CG/english/major-genital-infections-summary-23443460.html (2021, accessed 10 June 2025).

24. Bardach A, Alconada T, Palermo C, et al. Burden of Disease of Gonorrhoea in Latin America: Systematic Review and Meta-analysis. Infect Dis Ther 2023; 12: 1505–1525.

25. Zoni AC, González MA, Sjögren HW. Syphilis in the most at-risk populations in Latin America and the Caribbean: a systematic review. Int J Infect Dis 2013; 17: e84–e92.

26. Vallejo-Ortega MT, Gaitán Duarte H, Mello MB, et al. A systematic review of the prevalence of selected sexually transmitted infections in young people in Latin America. Rev Panam Salud Pública 2022; 46: 1.

27. Pan American Health Organization. Epidemiological Review of Syphilis in the Americas, December 2021, https://iris.paho.org/bitstream/handle/10665.2/56085/PAHOCDEHT220009_eng.pdf (2021).

28. UNAIDS. Honduras 2023 Country Factsheets, https://www.unaids.org/en/regionscountries/countries/honduras (2023).

29. Coelho LE, Torres TS, Veloso VG, et al. The Prevalence of HIV Among Men Who Have Sex With Men (MSM) and Young MSM in Latin America and the Caribbean: A Systematic Review. AIDS Behav 2021; 25: 3223–3237.

30. García PJ, Bayer A, Cárcamo CP. The Changing face of HIV in Latin America and the Caribbean. Curr HIV/AIDS Rep 2014; 11: 146–157.

31. Haddad L, Gadano A. HBV elimination in Central and South America: Current status and challenges. Clin Liver Dis; 23. Epub ahead of print January 2024. DOI: 10.1097/CLD.0000000000000226.

32. Coalition for Global Hepatitis Eliminiation. Accelerating Progress Toward Hepatitis Elimination in Latin America: Roundtable Discussion. 18 November 2021.

33. Laguna-Meraz S, Roman S, Jose-Abrego A, et al. A hospital-based study of the prevalence of HBV, HCV, HIV, and liver disease among a low-income population in West Mexico. Ann Hepatol 2022; 27: 100579.

34. Pan American Health Organization. Hepatitis B and C in the Spotlight A public health response in the Americas 2016. Washington, D.C., https://iris.paho.org/bitstream/handle/10665.2/31449/9789275119297-eng.pdf?sequence=5&isAllowed=y (2016).

35. World Health Organization. Hepatitis B Factsheet, https://www.who.int/news-room/fact-sheets/detail/hepatitis-b (2024).

36. World Health Organization. Hepatitis C Factsheet, https://www.who.int/news-room/fact-sheets/detail/hepatitis-c#:~:text=In%20settings%20with%20high%20HCV,prevent%20long%2Dterm%20liver%20damage. (2024).

37. Carnegie NB, Morris M. Size Matters: Concurrency and the Epidemic Potential of HIV in Small Networks. PLoS ONE 2012; 7: e43048.

38. Kim J-H. HIV Transmissions by Stage and Sex Role in Long-Term Concurrent Sexual Partnerships. Acta Biotheor 2015; 63: 33–54.

39. Reisner SL, Perez-Brumer A, Oldenburg CE, et al. Characterizing HIV risk among cisgender men in Latin America who report transgender women as sexual partners: HIV risk in Latin America men. Int J STD AIDS 2019; 30: 378–385.

40. World Health Organization. Guidelines for the management of asymptomatic sexually transmitted infections, https://iris.who.int/bitstream/handle/10665/381533/9789240104907-eng.pdf?sequence=1 (2025).

41. Raccagni AR, Castagna A, Nozza S. Gonorrhoea and chlamydia screening for asymptomatic people with HIV and HIV PrEP users: open issues. Lancet Infect Dis 2024; 24: e614–e615.

42. Kenyon C, Herrmann B, Hughes G, et al. Management of asymptomatic sexually transmitted infections in Europe: towards a differentiated, evidence-based approach. Lancet Reg Health - Eur 2023; 34: 100743.

43. Pai NP, Vadnais C, Denkinger C, et al. Point-of-Care Testing for Infectious Diseases: Diversity, Complexity, and Barriers in Low- And Middle-Income Countries. PLOS Med 2012; 9: e1001306.

44. Riegler AN, Larsen N, Amerson-Brown MH. Point-of-Care Testing for Sexually Transmitted Infections. Clin Lab Med 2023; 43: 189–207.

45. Lekodeba N, Snyman K, Nichols BE, et al. Cost, cost-effectiveness and budget impact analysis of near point-of-care GeneXpert testing for STIs in South Africa: leveraging current capacity to address high prevalence of Chlamydia trachomatis, Neisseria gonorrhoeae and Trichomonas vaginalis. 2025; 2024.12.13.24319006.

46. Gutiérrez JP, Trossero A. Socioeconomic inequalities in HIV knowledge, HIV testing, and condom use among adolescent and young women in Latin America and the Caribbean. Rev Panam Salud Pública 2021; 45: 1.

47. Muchomba FM, Chan C, El-Bassel N. Importance of Women's Relative Socioeconomic Status within Sexual Relationships in Communication about Safer Sex and HIV/STI Prevention. J Urban Health 2015; 92: 559–571.

48. Kerschberger B, Lekelem S, Daka M, et al. Mycoplasma genitalium infection in Eswatini amid syndromic case management: prevalence, coinfections, diagnostic challenges and treatment gaps. BMC Infect Dis 2025; 25: 547.
