# Supplementary figures and images for: STI prevalence and the integration of point-of-care nucleic acid amplification testing into STI diagnostic algorithms at a Médecins Sans Frontières key population clinic in San Pedro Sula, Honduras
Source: Front Reprod Health. 2026 Feb 23;8:1685453. doi: 10.3389/frph.2026.1685453 (PMC12968246; doi:10.3389/frph.2026.1685453)

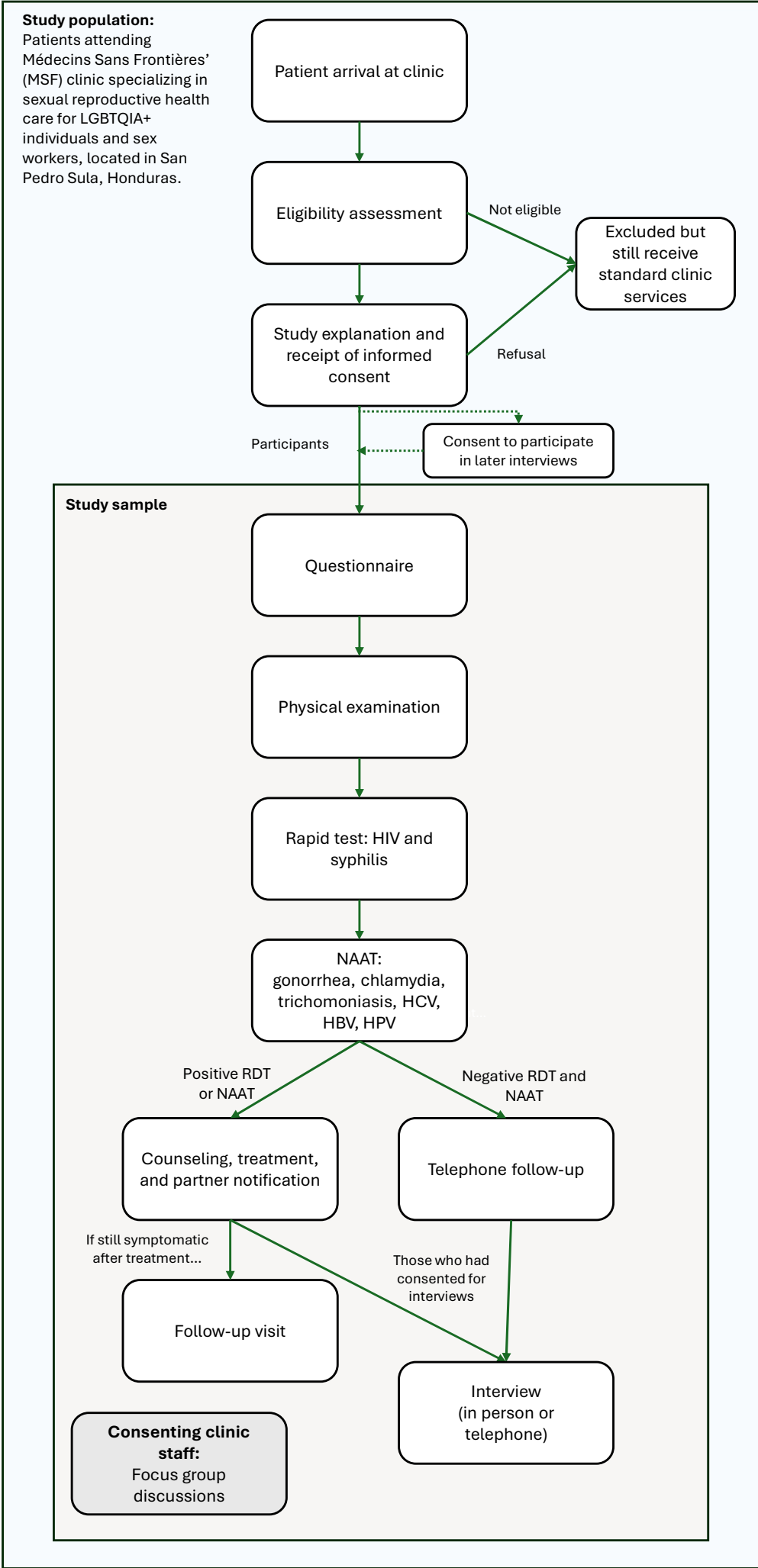

Supplement: Supplementary file 2 [file Image1.pdf]
